# 2-[(2-Chloro­quinolin-3-yl)(hy­droxy)meth­yl]acrylo­nitrile

**DOI:** 10.1107/S1600536813010155

**Published:** 2013-04-24

**Authors:** T. Anuradha, J. Srinivasan, P. R. Seshadri, M. Bakthadoss

**Affiliations:** a Post Graduate and Research Department of Physics, Agurchand Manmull Jain College, Chennai 600 114, India; b Department of Organic Chemistry, University of Madras, Guindy Campus, Chennai 600 025, India

## Abstract

In the title compound, C_13_H_9_ClN_2_O, the dihedral angle between the acrylo­nitrile C=C—CN plane and the quilonine ring system is 71.3 (2)°. In the crystal, mol­ecules are linked by O—H⋯N hydrogen bonds, forming chains along [01-1]. The chains are linked into a three-dimensional network through C—H⋯N inter­actions.

## Related literature

For the biological activity of quinoline and arcylo­nitrile compounds, see: Dutta *et al.* (2002 ▶); Ohsumi *et al.* (1998 ▶); Saczewski *et al.* (2004 ▶).
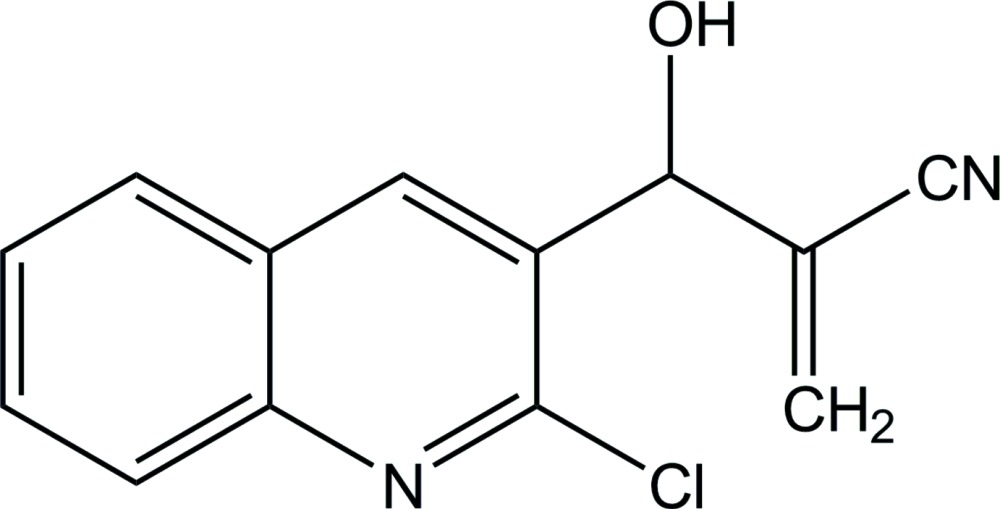


## Experimental

### 

#### Crystal data

C_13_H_9_ClN_2_O
*M* *_r_* = 244.67
Orthorhombic, 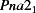

*a* = 12.2879 (12) Å
*b* = 9.6422 (11) Å
*c* = 10.3642 (12) Å
*V* = 1228.0 (2) Å^3^
*Z* = 4
Mo *K*α radiation
μ = 0.30 mm^−1^
*T* = 293 K
0.20 × 0.15 × 0.10 mm

#### Data collection

Bruker SMART APEXII area-detector diffractometer
Absorption correction: multi-scan (*SADABS*; Bruker, 2004 ▶) *T*_min_ = 0.943, *T*_max_ = 0.971
6334 measured reflections
2423 independent reflections
2144 reflections with *I* > 2σ(*I*)
*R*_int_ = 0.031

#### Refinement

*R*[*F* ^2^ > 2σ(*F* ^2^)] = 0.034
*wR*(*F* ^2^) = 0.090
*S* = 1.02
2423 reflections
156 parameters
1 restraint
H-atom parameters constrained
Δρ_max_ = 0.14 e Å^−3^
Δρ_min_ = −0.14 e Å^−3^
Absolute structure: Flack (1983 ▶), 819 Friedel pairs
Flack parameter: 0.02 (7)

### 

Data collection: *APEX2* (Bruker, 2008 ▶); cell refinement: *SAINT* (Bruker, 2008 ▶); data reduction: *SAINT*; program(s) used to solve structure: *SHELXS97* (Sheldrick, 2008 ▶); program(s) used to refine structure: *SHELXL97* (Sheldrick, 2008 ▶); molecular graphics: *ORTEP-3 for Windows* (Farrugia, 2012 ▶); software used to prepare material for publication: *SHELXL97*, *PLATON* (Spek, 2009 ▶) and *publCIF* (Westrip, 2010 ▶).

## Supplementary Material

Crystal structure: contains datablock(s) I, global. DOI: 10.1107/S1600536813010155/is5260sup1.cif

Structure factors: contains datablock(s) I. DOI: 10.1107/S1600536813010155/is5260Isup2.hkl

Supplementary material file. DOI: 10.1107/S1600536813010155/is5260Isup3.cml

Additional supplementary materials:  crystallographic information; 3D view; checkCIF report

## Figures and Tables

**Table 1:** Hydrogen-bond geometry (Å, °)

*D*—H⋯*A*	*D*—H	H⋯*A*	*D*⋯*A*	*D*—H⋯*A*
O1—H1⋯N1^i^	0.82	1.99	2.781 (2)	161
C10—H10⋯N2^ii^	0.98	2.57	3.385 (3)	140

Symmetry codes: (i) 
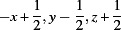
; (ii) 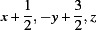.

**Fig. 1.**
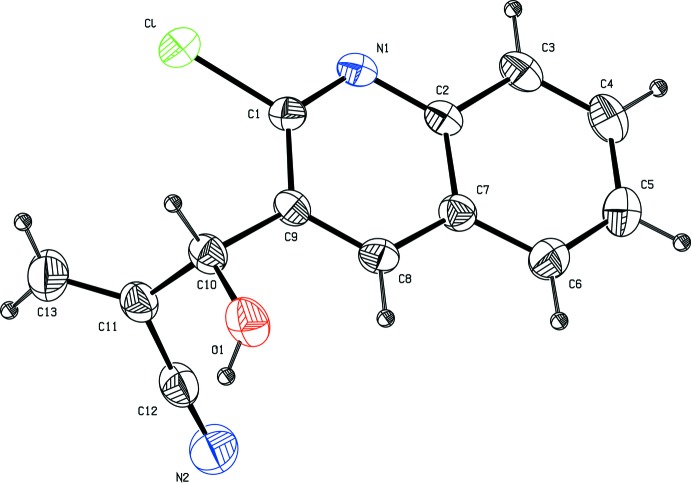
Molecular structure of the title compound, showing the atom-numbering scheme with 30% probability displacement ellipsoids. H atoms are shown as spheres of arbitrary radius.

**Fig. 2.**
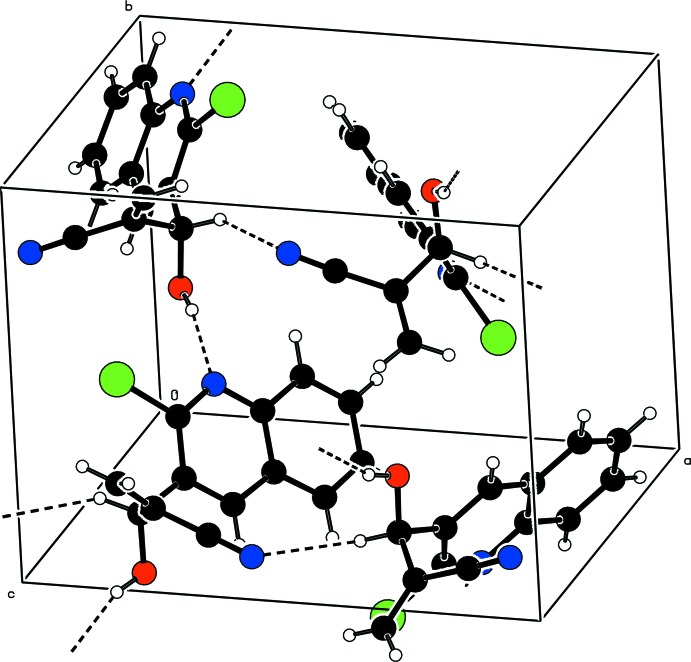
A view of packing of the molecules with hydrogen bonds (dashed lines).

## supplementary crystallographic information

### Comment

2-Chloro substituted quinolines are vital synthetic intermediates in the construction of a large number of linearly fusedtri- and tetra-cyclic quinolines studied for the DNA intercalating properties (Dutta *et al.*, 2002). Acrylonitrile derivatives have been shown to possess antitubercular and antitumour activities (Ohsumi *et al.*, 1998) and also in membranetechnology, synthesis and medicinal chemistry (Saczewski *et al.*, 2004).

In the title compound, the acrylonitrile (C11–C13/N2) and 2-chloroquilonine (C1–C9/N1/Cl) make a dihedral angle of 71.3 (2)°. Both the units are essentially planar with r.m.s. deviations of 0.012 and 0.008 Å, respectively. The hydroxyl group is anti-periplanar with the 2-chloroquilonine [torsion angle of O1—C10—C9—C1 = -155.10 (16)°] and -*syn* clinal with the acrylonitrile [torsion angle of O1—C10—C11—C13 = -52.3 (2)°]. The crystal structure is stabilized by intermolecular C—H···N and O—H···N interactions (Table 1).

### Experimental

A mixture of 2-chloroquinoline-3-carbaldehyde (0.1 g, 0.52 mmol), acrylonitrile (0.051 ml, 0.78 mmol), and DABCO (0.017 g, 0.15 mmol), was kept at room temperature for 3 days. After completion of the reaction (indicated by TLC), the reaction mixture was extracted with ethylacetate (3 × 15 ml). The combined organic layer subsequently washed with dil.HCl and dried over anhydrous Na_2_SO_4_. Solvent was evaporated under reduced pressure, crude product was obtained and purified by column chromatography eluting with 8% ethylacetate in hexane afforded the alcohol 2-[(2-chloroquinolin-3-yl)(hydroxy)methyl]acrylonitrile as a colourless solid.

### Refinement

Hydrogen atoms were positioned geometrically and allowed to ride on their parent atoms, with O—H = 0.82 Å and C—H = 0.93 or 0.98 Å, and with *U*_iso_(H) = 1.5*U*_eq_(O) and 1.2*U*_eq_(C).

### Crystal data

**Table:**

C_13_H_9_ClN_2_O	*F*(000) = 504
*M**_r_* = 244.67	*D*_x_ = 1.323 Mg m^−^^3^
Orthorhombic, *P**n**a*2_1_	Mo *K*α radiation, λ = 0.71073 Å
Hall symbol: P 2c -2n	Cell parameters from 2423 reflections
*a* = 12.2879 (12) Å	θ = 2.7–28.3°
*b* = 9.6422 (11) Å	µ = 0.30 mm^−^^1^
*c* = 10.3642 (12) Å	*T* = 293 K
*V* = 1228.0 (2) Å^3^	Block, colourless
*Z* = 4	0.20 × 0.15 × 0.10 mm

### Data collection

**Table:**

Bruker SMART APEXII area-detector diffractometer	2423 independent reflections
Radiation source: fine-focus sealed tube	2144 reflections with *I* > 2σ(*I*)
Graphite monochromator	*R*_int_ = 0.031
ω and φ scans	θ_max_ = 28.3°, θ_min_ = 2.7°
Absorption correction: multi-scan (*SADABS*; Bruker, 2004)	*h* = −15→16
*T*_min_ = 0.943, *T*_max_ = 0.971	*k* = −12→11
6334 measured reflections	*l* = −13→11

### Refinement

**Table:**

Refinement on *F*^2^	Hydrogen site location: inferred from neighbouring sites
Least-squares matrix: full	H-atom parameters constrained
*R*[*F*^2^ > 2σ(*F*^2^)] = 0.034	*w* = 1/[σ^2^(*F*_o_^2^) + (0.0445*P*)^2^ + 0.0827*P*] where *P* = (*F*_o_^2^ + 2*F*_c_^2^)/3
*wR*(*F*^2^) = 0.090	(Δ/σ)_max_ < 0.001
*S* = 1.02	Δρ_max_ = 0.14 e Å^−^^3^
2423 reflections	Δρ_min_ = −0.14 e Å^−^^3^
156 parameters	Extinction correction: *SHELXL97* (Sheldrick, 2008), Fc^*^=kFc[1+0.001xFc^2^λ^3^/sin(2θ)]^-1/4^
1 restraint	Extinction coefficient: 0.015 (3)
Primary atom site location: structure-invariant direct methods	Absolute structure: Flack (1983), 819 Friedel pairs
Secondary atom site location: difference Fourier map	Flack parameter: 0.02 (7)

### Special details

**Table:**

Geometry. All e.s.d.'s (except the e.s.d. in the dihedral angle between two l.s. planes) are estimated using the full covariance matrix. The cell e.s.d.'s are taken into account individually in the estimation of e.s.d.'s in distances, angles and torsion angles; correlations between e.s.d.'s in cell parameters are only used when they are defined by crystal symmetry. An approximate (isotropic) treatment of cell e.s.d.'s is used for estimating e.s.d.'s involving l.s. planes.
Refinement. Refinement of *F*^2^ against ALL reflections. The weighted *R*-factor *wR* and goodness of fit *S* are based on *F*^2^, conventional *R*-factors *R* are based on *F*, with *F* set to zero for negative *F*^2^. The threshold expression of *F*^2^ > σ(*F*^2^) is used only for calculating *R*-factors(gt) *etc*. and is not relevant to the choice of reflections for refinement. *R*-factors based on *F*^2^ are statistically about twice as large as those based on *F*, and *R*- factors based on ALL data will be even larger.

### Fractional atomic coordinates and isotropic or equivalent isotropic displacement parameters (Å^2^)

**Table:**

	*x*	*y*	*z*	*U*_iso_*/*U*_eq_	
Cl	0.29993 (4)	1.02688 (6)	0.48433 (7)	0.06601 (17)	
O1	0.22815 (13)	0.60309 (15)	0.62021 (17)	0.0682 (4)	
H1	0.2656	0.5770	0.6811	0.102*	
N1	0.16299 (12)	0.95206 (16)	0.30888 (16)	0.0476 (3)	
N2	−0.02036 (17)	0.7266 (3)	0.7546 (3)	0.0870 (7)	
C1	0.20463 (12)	0.91298 (18)	0.41795 (18)	0.0438 (4)	
C2	0.08750 (12)	0.86715 (18)	0.25212 (17)	0.0441 (4)	
C3	0.04132 (16)	0.9065 (2)	0.1326 (2)	0.0574 (5)	
H3	0.0617	0.9897	0.0941	0.069*	
C4	−0.03276 (18)	0.8230 (2)	0.0741 (2)	0.0643 (5)	
H4	−0.0628	0.8496	−0.0045	0.077*	
C5	−0.06450 (18)	0.6974 (3)	0.1306 (2)	0.0698 (6)	
H5	−0.1157	0.6418	0.0894	0.084*	
C6	−0.02145 (18)	0.6556 (2)	0.2451 (2)	0.0637 (5)	
H6	−0.0428	0.5716	0.2813	0.076*	
C7	0.05581 (13)	0.73997 (18)	0.30916 (19)	0.0467 (4)	
C8	0.10462 (14)	0.70438 (18)	0.42751 (19)	0.0500 (4)	
H8	0.0850	0.6219	0.4678	0.060*	
C9	0.18017 (11)	0.78848 (16)	0.4845 (2)	0.0427 (3)	
C10	0.23288 (14)	0.7498 (2)	0.6112 (2)	0.0499 (4)	
H10	0.3091	0.7796	0.6106	0.060*	
C11	0.17479 (15)	0.8165 (2)	0.7227 (2)	0.0531 (4)	
C12	0.06476 (15)	0.7679 (2)	0.7430 (2)	0.0596 (5)	
C13	0.2182 (2)	0.9086 (3)	0.8014 (3)	0.0818 (8)	
H13A	0.1780	0.9428	0.8704	0.098*	
H13B	0.2890	0.9394	0.7878	0.098*	

### Atomic displacement parameters (Å^2^)

**Table:**

	*U*^11^	*U*^22^	*U*^33^	*U*^12^	*U*^13^	*U*^23^
Cl	0.0630 (2)	0.0706 (3)	0.0645 (3)	−0.0222 (2)	−0.0026 (3)	0.0007 (3)
O1	0.0915 (10)	0.0563 (8)	0.0569 (9)	0.0187 (7)	−0.0204 (8)	0.0116 (7)
N1	0.0539 (7)	0.0462 (8)	0.0426 (8)	−0.0018 (6)	0.0047 (7)	0.0098 (6)
N2	0.0582 (10)	0.1206 (18)	0.0821 (17)	−0.0155 (11)	0.0037 (10)	−0.0078 (14)
C1	0.0430 (7)	0.0452 (8)	0.0432 (9)	−0.0038 (6)	0.0047 (7)	0.0019 (7)
C2	0.0484 (7)	0.0457 (8)	0.0382 (9)	0.0043 (6)	0.0028 (7)	0.0058 (7)
C3	0.0692 (10)	0.0596 (11)	0.0433 (11)	0.0076 (9)	0.0002 (9)	0.0132 (9)
C4	0.0708 (11)	0.0765 (14)	0.0456 (11)	0.0133 (10)	−0.0115 (10)	0.0020 (10)
C5	0.0709 (11)	0.0768 (14)	0.0616 (14)	−0.0064 (10)	−0.0156 (12)	−0.0068 (12)
C6	0.0722 (11)	0.0581 (11)	0.0609 (14)	−0.0126 (9)	−0.0076 (11)	0.0040 (10)
C7	0.0521 (8)	0.0450 (9)	0.0430 (10)	0.0008 (7)	−0.0004 (8)	0.0041 (7)
C8	0.0586 (8)	0.0426 (8)	0.0487 (10)	−0.0024 (7)	−0.0025 (8)	0.0123 (7)
C9	0.0456 (6)	0.0449 (8)	0.0376 (8)	0.0054 (5)	−0.0010 (8)	0.0059 (8)
C10	0.0493 (8)	0.0561 (10)	0.0443 (9)	0.0061 (7)	−0.0079 (8)	0.0077 (8)
C11	0.0526 (8)	0.0632 (11)	0.0436 (10)	−0.0009 (8)	−0.0064 (8)	0.0053 (9)
C12	0.0574 (10)	0.0746 (13)	0.0469 (11)	0.0031 (9)	−0.0044 (9)	−0.0002 (9)
C13	0.0761 (13)	0.102 (2)	0.0678 (16)	−0.0144 (13)	0.0020 (13)	−0.0238 (16)

### Geometric parameters (Å, º)

**Table:**

Cl—C1	1.7466 (17)	C5—H5	0.9300
O1—C10	1.419 (3)	C6—C7	1.416 (3)
O1—H1	0.8200	C6—H6	0.9300
N1—C1	1.297 (2)	C7—C8	1.408 (3)
N1—C2	1.370 (2)	C8—C9	1.367 (2)
N2—C12	1.125 (3)	C8—H8	0.9300
C1—C9	1.417 (2)	C9—C10	1.511 (3)
C2—C3	1.414 (3)	C10—C11	1.504 (3)
C2—C7	1.416 (2)	C10—H10	0.9800
C3—C4	1.358 (3)	C11—C13	1.318 (3)
C3—H3	0.9300	C11—C12	1.447 (3)
C4—C5	1.401 (4)	C13—H13A	0.9300
C4—H4	0.9300	C13—H13B	0.9300
C5—C6	1.360 (3)		
			
C10—O1—H1	109.5	C8—C7—C2	117.24 (16)
C1—N1—C2	117.89 (15)	C6—C7—C2	119.12 (18)
N1—C1—C9	125.93 (16)	C9—C8—C7	121.42 (16)
N1—C1—Cl	115.16 (13)	C9—C8—H8	119.3
C9—C1—Cl	118.90 (14)	C7—C8—H8	119.3
N1—C2—C3	119.17 (16)	C8—C9—C1	115.88 (17)
N1—C2—C7	121.64 (16)	C8—C9—C10	121.34 (16)
C3—C2—C7	119.19 (17)	C1—C9—C10	122.78 (15)
C4—C3—C2	120.07 (18)	O1—C10—C11	110.90 (18)
C4—C3—H3	120.0	O1—C10—C9	106.62 (16)
C2—C3—H3	120.0	C11—C10—C9	111.04 (14)
C3—C4—C5	120.80 (19)	O1—C10—H10	109.4
C3—C4—H4	119.6	C11—C10—H10	109.4
C5—C4—H4	119.6	C9—C10—H10	109.4
C6—C5—C4	120.8 (2)	C13—C11—C12	120.5 (2)
C6—C5—H5	119.6	C13—C11—C10	124.89 (19)
C4—C5—H5	119.6	C12—C11—C10	114.60 (17)
C5—C6—C7	120.0 (2)	N2—C12—C11	177.2 (3)
C5—C6—H6	120.0	C11—C13—H13A	120.0
C7—C6—H6	120.0	C11—C13—H13B	120.0
C8—C7—C6	123.64 (17)	H13A—C13—H13B	120.0
			
C2—N1—C1—C9	0.7 (3)	C2—C7—C8—C9	−0.6 (3)
C2—N1—C1—Cl	−179.85 (13)	C7—C8—C9—C1	0.7 (3)
C1—N1—C2—C3	−179.50 (16)	C7—C8—C9—C10	−179.68 (17)
C1—N1—C2—C7	−0.5 (2)	N1—C1—C9—C8	−0.8 (3)
N1—C2—C3—C4	179.27 (18)	Cl—C1—C9—C8	179.74 (13)
C7—C2—C3—C4	0.2 (3)	N1—C1—C9—C10	179.58 (17)
C2—C3—C4—C5	0.0 (3)	Cl—C1—C9—C10	0.2 (2)
C3—C4—C5—C6	−0.4 (4)	C8—C9—C10—O1	25.3 (2)
C4—C5—C6—C7	0.5 (4)	C1—C9—C10—O1	−155.10 (16)
C5—C6—C7—C8	−179.8 (2)	C8—C9—C10—C11	−95.6 (2)
C5—C6—C7—C2	−0.3 (3)	C1—C9—C10—C11	84.0 (2)
N1—C2—C7—C8	0.4 (2)	O1—C10—C11—C13	125.4 (2)
C3—C2—C7—C8	179.43 (17)	C9—C10—C11—C13	−116.3 (3)
N1—C2—C7—C6	−179.12 (18)	O1—C10—C11—C12	−52.3 (2)
C3—C2—C7—C6	−0.1 (3)	C9—C10—C11—C12	66.1 (2)
C6—C7—C8—C9	178.9 (2)		

### Hydrogen-bond geometry (Å, º)

**Table:**

*D*—H···*A*	*D*—H	H···*A*	*D*···*A*	*D*—H···*A*
O1—H1···N1^i^	0.82	1.99	2.781 (2)	161
C10—H10···N2^ii^	0.98	2.57	3.385 (3)	140

Symmetry codes: (i) −*x*+1/2, *y*−1/2, *z*+1/2; (ii) *x*+1/2, −*y*+3/2, *z*.
